# Association Between the Triglyceride-Glucose Index and Serum Uric Acid to High-Density Lipoprotein (HDL) Cholesterol Ratio in Type 2 Diabetes Mellitus in Gabes City, Tunisia

**DOI:** 10.7759/cureus.68235

**Published:** 2024-08-30

**Authors:** Hamida Kwas, Hayfa Rajhi, Harish Rangareddy

**Affiliations:** 1 Pulmonology, University of Sfax, Faculty of Medicine of Sfax, University Hospital of Gabès, Gabès, TUN; 2 Analysis Laboratory Research, University Hospital of Gabès, Gabès, TUN; 3 Biochemistry, Haveri Institute of Medical Sciences, Haveri, IND

**Keywords:** uric acid to hdlc ratio, cardiovascular disease, types 2 diabetes, resistance to insulin, triglyceride-glucose index (tyg)

## Abstract

Background

Type 2 diabetes mellitus (T2DM) is a significant risk factor for cardiovascular diseases (CVD). The triglyceride-glucose index (TyGi) is a novel biomarker for insulin resistance, strongly linked to CVD. Elevated serum uric acid levels and the uric acid to high-density lipoprotein cholesterol ratio (UHR) are emerging as markers of metabolic syndrome and cardiovascular risk in T2DM. This study aimed to explore the association between the TyGi and UHR in T2DM patients.

Objectives

The aim of this study is to compare metabolic parameters in T2DM patients and assess the association between the TyGi and serum UHR.

Methodology

A cross-sectional case-control study was conducted at the University Hospital of Gabes, Gabes City, Tunisia with 50 T2DM patients and 50 gender-matched healthy controls. Inclusion criteria included adults aged 30-75 years with a confirmed diagnosis of T2DM on stable medication for at least three months. Exclusion criteria included other types of diabetes, significant liver or kidney disease, recent cardiovascular events, endocrine disorders, and substance abuse. Metabolic and biochemical parameters, including fasting blood sugar, postprandial blood sugar, glycosylated hemoglobin, lipid profile, and renal function, were measured. The TyGi and serum UHR were calculated and analyzed for correlations.

Results

T2DM patients exhibited significantly higher fasting blood sugar, postprandial blood sugar, glycosylated hemoglobin, TyGi, and serum UHR compared to controls, indicating impaired glycemic control and adverse lipid profiles. The UHR showed a positive correlation with a strong negative correlation with HDL and a positive correlation with uric acid levels. The linear regression analysis indicated a weak positive trend between the TyGi and serum UHR, although not statistically significant.

Conclusion

This study underscores the importance of the TyGi and serum UHR as biomarkers for evaluating metabolic and cardiovascular risk in T2DM. Further research is needed to explore their combined utility in clinical practice for early detection and management of cardiovascular complications in diabetic patients.

## Introduction

Type 2 diabetes mellitus (T2DM) is a major risk factor for cardiovascular diseases (CVD), especially myocardial infarction (MI) [1]. The triglyceride-glucose index (TyGi) index has emerged as a reliable alternative biomarker for insulin resistance (IR) [2]. Recent studies have provided strong statistical evidence indicating that the TyGi is linked to the development and prognosis of CVD [3-5]. Understanding the metabolic differences between T2DM patients with and without MI can help identify potential biomarkers and therapeutic targets to prevent cardiac events in diabetic patients. Elevated serum uric acid levels have been linked to hypertension and T2DM. The uric acid to high-density lipoprotein cholesterol (HDLc) ratio (UHR) has been suggested as an effective diagnostic tool for metabolic syndrome in T2DM patients [6]. Additionally, the UHR serves as a marker with a notable correlation to fasting blood glucose (FBS) and glycosylated hemoglobin (HbA1c) levels for evaluating the management of T2DM [7]. The UHR is a marker that rises in inflammatory states [8]. It has been proposed to use the UHR as an efficient method for diagnosing CVD risk in non-diabetic subjects in preclinical stages [9]. Due to the paucity of research on the connection between the TyGi and UHR, this study aimed to establish the association between these metabolic parameters. This study aimed to compare various metabolic parameters in T2DM patients and assess the association of the TyGi with the UHR.

## Materials and methods

Study design

This cross-sectional case-control study was conducted employing purposive sampling to select previously diagnosed cases of T2DM patients attending follow-up visits to the Pulmonology department and the Internal Medicine department at the University Hospital of Gabes, Tunisia. The hospital primarily caters to patients from the urban and surrounding rural areas within the Gabes Governorate, a region known for its mixed population demographics and varying socio-economic conditions in Tunisia. These patients were apparently healthy, with the only significant co-morbidity being T2DM. Although these departments generally manage a variety of conditions, the inclusion and exclusion criteria ensured that participants did not have active illnesses that could confound the study results. Only those patients who were attending routine follow-up visits, without any acute or chronic conditions requiring treatment other than T2DM, were included. This careful selection was intended to minimize potential confounding variables, thereby allowing the study to focus on the specific relationship between T2DM and the variables of interest.

The Institutional Ethics Committee approval for the study protocol was obtained and access to the database was strictly limited to analytical purposes, with personal information remaining inaccessible. Laboratory reports of T2DM patients from the Central Diagnostic Laboratory were gathered, ensuring data anonymization procedures were followed rigorously.

Sample size

The sample size was calculated considering the mean differences of serum UHR measured in a study by Yazdi et al. [10].

n = (Z_α/2_+Z_β_)^2^ *2*σ^2 ^/ d^2^

where Z_α/2_ is the critical value of the normal distribution at α/2 (for a confidence level of 95%, α is 0.05 and the critical value is 1.96), Z_β_ is the critical value of the normal distribution at β (for a power of 80%, β is 0.2 and the critical value is 0.84), σ^2^ is the population variance, and d is the mean difference. The sample size was estimated to be 36 in each group. Fifty T2DM patients and 50 gender-matched healthy controls were enrolled in the study.

The study's inclusion criteria were as follows: adults aged 30 to 75 years with a confirmed diagnosis of T2DM based on the American Diabetes Association criteria, who had been on a stable anti-diabetic medication regimen for at least three months, and who had not had any recent hospitalizations for acute illnesses in the three months prior to enrollment. Participants were excluded if they had type 1 diabetes or any other type of diabetes, including gestational diabetes. Exclusion criteria included having an estimated glomerular filtration rate (eGFR) of less than 30 mL/min/1.73 m², chronic liver disease and a history of MI, congestive heart failure, or other serious cardiovascular problems within the last six months, as well as the existence of thyroid or other endocrine disorders impacting metabolism. Pregnant or breastfeeding women were also excluded from the study.

In this study, we assessed a range of biochemical and anthropometric parameters to evaluate metabolic health in diabetic patients and controls. FBS and postprandial blood sugar (PPBS) levels were measured using standard enzymatic methods, while HbA1C levels were determined through high-performance liquid chromatography to assess long-term glycemic control. Lipid profiles, including total cholesterol (TC), triglycerides (TyG), high-density lipoprotein (HDL), and low-density lipoprotein (LDL), were measured using enzymatic colorimetric assays. The TyGi index, an indicator of insulin resistance, was calculated using the formula: TyGi = ln [TyG (mg/dL) × FBS (mg/dL)/2] (where ln represents the natural logarithm) [11]. Body mass index (BMI) was calculated as weight (kg) divided by height (m²). Renal function was evaluated by calculating the eGFR using the Chronic Kidney Disease Epidemiology Collaboration equation [12]. Uric acid levels were determined enzymatically. The UHR, a marker of cardiovascular risk, was computed by dividing serum uric acid levels by HDL cholesterol levels.

Statistical analysis

Data collected was tabulated and entered in Microsoft Excel (Microsoft Corporation, Redmond, USA). The Kolmogorov-Smirnov test was employed to detect the normal distribution of data, leading to the application of appropriate parametric statistical tests which included the independent 't' test and Pearson's correlation analysis. Statistical analysis was performed using IBM SPSS Statistics for Windows, Version 16 (Released 2007; IBM Corp., Armonk, New York, United States), and significance was set at p < 0.05.

## Results

The study involved 50 cases of individuals diagnosed with T2DM. The age of participants ranged from 31 to 75 years, with a mean age of approximately 53 years. The gender distribution included 29 males and 21 females. The duration of diabetes among the participants varied widely, from 1 year to 20 years, with a mean duration of about nine years. This diverse range in age and duration provides a comprehensive overview of the demographic characteristics of the study population, reflecting a typical cross-section of individuals affected by T2DM as depicted in Table 1.

**Table 1:** Demographics of study participants T2DM: Type 2 diabetes mellitus

Characteristic	Cases (n=50)	Controls (n=50)
Gender		
Males	29	29
Females	21	21
Age (years)		
Mean±SD	54.68±10.55	52.50±9.60
Males	55.34±11.67	50.34±9.11
Females	55.47±9.67	53.76±8.98
Duration of T2DM (years)		
Mean±SD	7.94±5.38	NA
Males	8.48±5.33	NA
Females	7.19±5.5	NA

Furthermore, a comprehensive analysis was carried out to investigate the association between the TyGi and the serum UHR in patients with T2DM. This analysis involved both the independent t-test to compare cases and controls and Pearson’s correlation to explore relationships among various metabolic and biochemical parameters.

In the comparison between T2DM and controls, several significant differences were observed as depicted in Table 2.

**Table 2:** Comparison of various metabolic parameters by the independent t-test Significance level: **p < 0.01, *p < 0.05 BMI: Body Mass Index; FBS: Fasting Blood Sugar; PPBS: Post Prandial Blood Sugar; HbA1C: Glycosylated Hemoglobin; TC: Total Cholesterol; TyG: Triglycerides; TyGi: Triglyceride-Glucose Index; TyGiBMI: Triglyceride-Glucose-Body Mass Index; HDLc: High-Density Lipoprotein Cholesterol; LDLc: Low-Density Lipoprotein Cholesterol; UA: Uric Acid; UHR: Serum Uric Acid to HDLc Ratio; eGFR: Estimated Glomerular Filtration Rate

Parameter	T2DM (n=50) Mean ± SD	Controls (n=50) Mean ± SD	t-value	df	p-value
BMI (kg/m^2^)	24.60 ± 4.10	24.34 ± 4.09	0.317	98	0.752
FBS (mg/dL)	143.32 ± 72.11	103.56 ± 18.03	3.783	98	0.001**
PPBS (mg/dL)	237.20 ± 93.83	115.36 ± 24.10	8.893	98	0.001**
HbA1C (%)	9.56 ± 1.82	5.47 ± 0.44	15.450	98	0.001**
TC (mg/dL)	145.66 ± 51.72	165.82 ± 53.81	-1.910	98	0.059
TyG (mg/dL)	145.54 ± 69.30	129.68 ± 76.34	1.088	98	0.279
TyGi	9.03 ± 0.76	8.68 ± 0.54	2.665	98	0.009**
TyGiBMI	221.72 ± 39.31	211.26 ± 38.65	1.342	98	0.183
HDLc (mg/dL)	34.34 ± 14.05	41.98 ± 14.20	-2.704	98	0.008**
LDLc (mg/dL)	82.96 ± 43.03	99.82 ± 43.26	-1.954	98	0.054
UA (mg/dL)	5.19 ± 1.09	4.59 ± 1.00	2.858	98	0.005**
UHR (%)	18.80 ± 11.70	12.16 ± 5.16	3.669	98	0.001**
eGFR (mL/min/1.73m^2^)	95.88 ± 56.99	104.26 ± 24.81	-0.953	98	0.343

Diabetics exhibited markedly poorer glycemic control, with significantly higher FBS levels (143.32 ± 72.11 vs. 103.56 ± 18.03 mg/dL, p < 0.001), PPBS levels (237.20 ± 93.83 vs. 115.36 ± 24.10 mg/dL, p < 0.001), and HbA1C levels (9.56 ± 1.82 vs. 5.47 ± 0.44%, p < 0.001) compared to controls. These findings highlight the impaired glycemic regulation in the diabetic cohort.

Lipid metabolism also differed significantly between the groups. Diabetic patients had lower HDL cholesterol levels (34.34 ± 14.05 vs. 41.98 ± 14.20 mg/dL, p = 0.008) and a higher TyGi (9.03 ± 0.76 vs. 8.68 ± 0.54, p = 0.009), indicating an adverse lipid profile. Additionally, the UHR was substantially higher in diabetics (18.80 ± 11.70 vs. 12.16 ± 5.16, p < 0.001), suggesting increased cardiovascular risk. Serum uric acid levels were also elevated in the diabetic group (5.19 ± 1.09 vs. 4.59 ± 1.00 mg/dL, p = 0.005).

Although the eGFR values were lower in diabetics, suggesting potential kidney function impairment, the difference was not statistically significant (p = 0.343). No significant differences were found in BMI, TC, TyG, low-density lipoprotein cholesterol (LDLc) and TyGiBMI. These results underscore the significant metabolic disturbances in diabetic patients, particularly concerning glycemic control and lipid metabolism, which may contribute to their elevated cardiovascular risk.

The Pearson correlation analysis revealed several significant relationships among the measured parameters as shown in Table 3.

**Table 3:** Significant Pearson correlation coefficients for biochemical parameters Significance level: **p < 0.01, *p < 0.05 BMI: Body Mass Index; FBS: Fasting Blood Sugar; PPBS: Post Prandial Blood Sugar; HbA1C: Glycosylated Hemoglobin; TC: Total Cholesterol; TyG: Triglycerides; TyGi: Triglyceride-Glucose Index; TyGiBMI: Triglyceride-Glucose-Body Mass Index; HDLc: High-Density Lipoprotein Cholesterol; LDLc: Low-Density Lipoprotein Cholesterol; UA: Uric Acid; UHR: Serum Uric Acid to HDLc Ratio

Variable 1	Variable 2	Pearson Correlation	Significance (p-value)
BMI	TyGiBMI	0.887**	0.000
FBS	PPBS	0.794**	0.000
FBS	TyGi	0.787**	0.000
PPBS	TyGi	0.738**	0.000
HbA1C	TyG	0.471**	0.001
TC	HDLc	0.536**	0.000
TC	LDLc	0.957**	0.000
UHR	HDLc	-0.786**	0.000
UHR	UA	0.403**	0.004
HDLc	LDLc	0.388**	0.005
BMI	HbA1C	0.336*	0.017
FBS	TyG	0.297*	0.036
PPBS	TyG	0.338*	0.016
TC	TyGi	0.317*	0.025
UHR	TyG	0.301*	0.034

A very strong positive correlation was observed between BMI and TyGiBMI (r = 0.887, p = 0.000), indicating that higher BMI is strongly associated with higher TyGiBMI. There was a strong positive correlation between FBS and PPBS (r = 0.794, p = 0.000) and between FBS and TyGi (r = 0.787, p = 0.000), suggesting that FBS levels are closely linked with postprandial blood sugar levels and the TyGi. Similarly, HbA1C showed a significant positive correlation with TyG (r = 0.471, p = 0.001), indicating that higher HbA1C levels are associated with higher TyG values. TC demonstrated a significant positive correlation with both HDL (r = 0.536, p = 0.000) and LDL (r = 0.957, p = 0.000), reflecting the interdependence of lipid parameters. The UHR exhibited a strong negative correlation with HDL (r = -0.786, p = 0.000) and a positive correlation with uric acid (r = 0.403, p = 0.004), suggesting that an increased UHR is associated with lower HDL levels and higher uric acid levels. HDL also showed a positive correlation with LDL (r = 0.388, p = 0.005), indicating that these lipid fractions tend to rise together.

**Figure 1:**
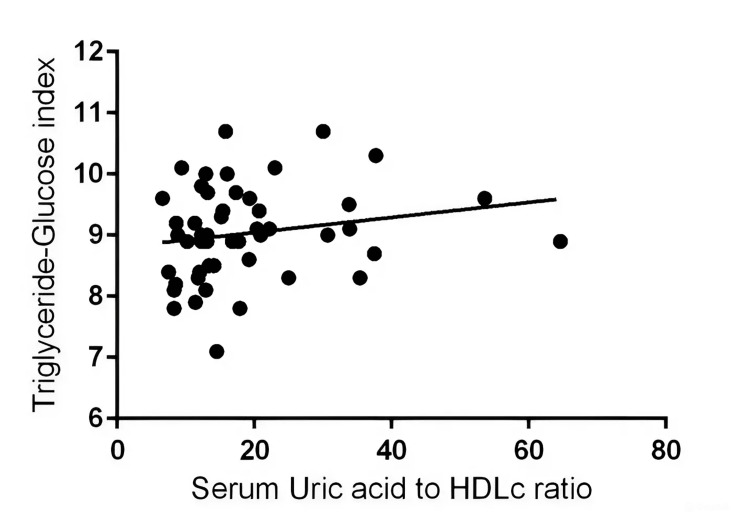
Linear regression analysis of the triglyceride-glucose index and serum uric acid to HDLc ratio HDLc: High-Density Lipoprotein Cholesterol

The linear regression analysis aimed to explore the relationship between the TyGi and the UHR. The results indicate that the best-fit line for this relationship has a slope of 0.01230, meaning for each unit increase in the TyGi, the UHR increases by approximately 0.01230 units Y = 0.01230*X + 8.799 as depicted in Figure 1.

However, the standard error of the slope (0.009201) and the 95% confidence interval for the slope (-0.006219 to 0.03082) suggest considerable variability around this estimate. The R square value of 0.03589 implies that only 3.589% of the variance in the UHR can be explained by the TyGi, indicating a very weak linear relationship between these two variables. The F-value of 1.787 with a p-value of 0.1876 indicates that the slope is not significantly different from zero. This suggests that while the regression analysis indicates a slight positive trend between the TyGi and the UHR, the relationship is not statistically significant, and the TyGi does not appear to be a strong predictor of the UHR in this study population.

## Discussion

In this study, we investigated the association between the TyGi and the serum UHR in patients with T2DM. Our findings provide important insights into the potential role of these biomarkers in assessing metabolic and cardiovascular risk in the population of Gabes in Tunisia. 

The significant correlations observed between the TyGi and various metabolic parameters underline its utility as a surrogate marker for insulin resistance. The TyGi showed a strong positive correlation with FBS and PPBS, consistent with previous studies indicating its effectiveness in predicting impaired glycemic control [13,14]. In a study by Son et al., the data from 9730 adults with or without metabolic syndrome (MetS) at baseline, 6091 adults without MetS who were followed as part of the Korean Genome and Epidemiology Study were analyzed and the TyGi was found to be superior to HOMA-IR for predicting MetS [15]. Additionally, the TyGi was significantly associated with HbA1c levels, further emphasizing its relevance in monitoring long-term glucose regulation in T2DM patients. This finding is at par with the observations of Selvi et al. that in the group with poor glycemic control, TyGi was significantly higher and correlated positively with HbA1c [16].

In addition to DM, IR is a key feature of obesity, hypertension, and dyslipidemia (characterized by hypertriglyceridemia and reduced HDL, and these MetS components are recognized as independent risk factors for CVD [17]. The TyGi, a valuable surrogate marker of IR, has increasingly been associated with the onset of CVD and adverse outcomes [18,19]. In a nested case-control study by Jin et al., involving T2DM patients with stable CAD, Kaplan-Meier analysis revealed that patients in the upper tertiles of the TyGi and hemoglobin glycation index (HGI) had significantly lower event-free survival. Both the TyGi and HGI were linked to an increased risk of major adverse cardiovascular and cerebrovascular events after adjusting for confounding factors. Additionally, incorporating the TyGi into the Cox model improved the predictive accuracy, whereas the inclusion of the HGI did not significantly enhance the model [20]. In a study by Che et al., 403,335 participants from the UK Biobank, free from CVD at baseline, were analyzed for the TyGi and TyG/HDL-C ratio. Cox models assessed the association between these markers and incident CVD. Mediation analyses revealed that dyslipidemia, type 2 diabetes, and hypertension accounted for 45.8%, 27.0%, and 15.0% of the TyGi's association with CVD, respectively, and 40.0%, 11.8%, and 13.3% of the TyG/HDL-C ratio's association with CVD, respectively [21]. Wang et al. observed that an elevated TyGi is linked to a higher risk of multi-vessel coronary artery disease (CAD), suggesting that the TyGi, as a measure of insulin resistance, could be a valuable predictor of CAD severity, particularly in individuals with pre-diabetes [22].

Our analysis revealed a significant positive correlation between the UHR and serum uric acid levels, underscoring the influence of elevated uric acid on this ratio. Elevated serum uric acid has been consistently associated with hypertension and T2DM, suggesting a potential link between hyperuricemia and metabolic syndrome [23,24]. The use of the UHR as a diagnostic tool for metabolic syndrome in T2DM patients is supported by our findings, which align with previous research highlighting its association with FBS and HbA1c levels [6,7].

In a study by Park et al., data from 16,455 participants without diabetes from the Health Risk Assessment Study (HERAS) and Korean Health Insurance Review and Assessment (HIRA) were analyzed. The findings revealed that high UHR values were positively associated with incident ischemic heart disease (IHD) in Koreans without diabetes. This suggests that an increased UHR may be a useful measure for assessing cardiovascular risk in the preclinical stage [9]. In a study by Liu et al., the relationship between the UHR and mortality was examined in 1953 eligible incident patients undergoing peritoneal dialysis. Over a median follow-up of 61.3 months, 567 patients died, with 48.3% due to cardiovascular causes. The mean baseline UHR was 16.4 ± 6.7%. The highest quartile UHR had hazard ratios of 1.35 for all-cause mortality and 1.46 for cardiovascular mortality compared to the second quartile [25]. The findings of this study are in line with previous reports that an elevated UHR is associated with vascular pathologies. In a study by Mansiroglu et al., patients with coronary artery fistula have been shown to have higher UHR levels compared to individuals with normal coronary arteries [26]. This reinforces the potential of the UHR as a marker for vascular health and its utility in clinical settings for early detection and management of cardiovascular complications in T2DM patients.

**Figure 2:**
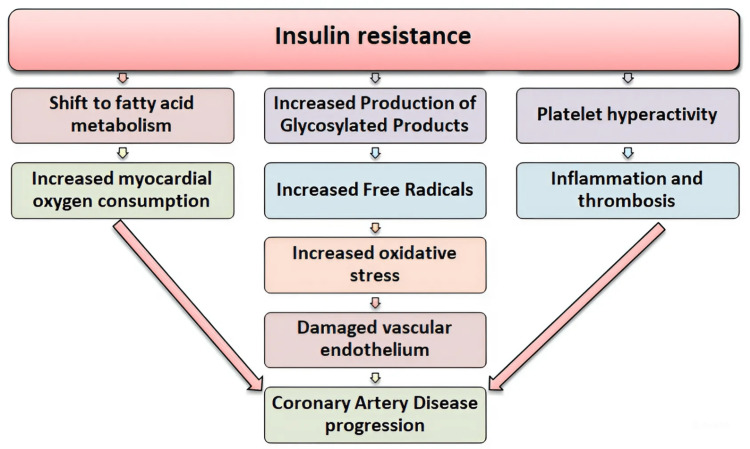
Mechanisms of insulin resistance leading to cardiovascular events Source: Image created by Harish Rangareddy is marked with CC0 1.0 Universal (https://creativecommons.org/publicdomain/zero/1.0/) Data from [2,27]

Both the TyGi and UHR are observed to be increased in IR and the possible mechanisms of IR leading to cardiovascular events are depicted in Figure 2.

This study showed that T2DM patients had significantly higher FBS, PPBS, HbA1c, and serum uric acid levels compared to the control group. These findings reflect the characteristic metabolic disturbances in diabetes, including poor glycemic control and increased cardiovascular risk. The elevated UHR in T2DM patients further supports the hypothesis that this ratio can serve as a marker for metabolic dysregulation and cardiovascular risk assessment. The TyGi was also significantly correlated with lipid parameters, including TC and LDLc. This suggests that dyslipidemia in T2DM patients is closely linked to IR and glucose metabolism.

Limitations of the study

The study was conducted within a single geographical region of Gabes in Tunisia, where dietary practices are relatively uniform due to shared cultural and socioeconomic factors. While individual dietary variations were not explicitly controlled for, the homogeneity of the population's diet within this region reduces the likelihood of significant dietary differences acting as a confounding factor. This consistency in diet across the study population supports the reliability of the observed associations between blood sugar, uric acid levels, and the co-morbidity of T2DM. The study’s findings should be interpreted within this regional context, as they may reflect the specific health challenges and resources available in the Gabes region.

This study concentrated on the analysis of metabolic biomarkers, such as FBS, PPBS, HbA1c, and serum uric acid levels, without extending the scope to clinical outcomes like cardiovascular events, renal function, or diabetic complications. While these biomarkers provide valuable insights into metabolic disturbances, integrating clinical outcomes would have allowed for a more comprehensive understanding of the clinical implications and potential long-term risks associated with these metabolic alterations. Future studies might expand on these findings by comparing them with data from other regions to assess regional variations in T2DM management and outcomes.

## Conclusions

The TyGi and UHR are valuable biomarkers for assessing metabolic and cardiovascular risk in T2DM patients. Their significant associations with key metabolic parameters, including FBS, PPBS, HbA1c, and lipid profiles, highlight their potential utility in clinical practice. Further studies are warranted to explore the underlying mechanisms and validate these findings in larger, diverse populations. The integration of these biomarkers into routine clinical assessments could enhance the early detection and management of metabolic and cardiovascular complications in T2DM patients.
